# Decarboxylase mediated oxalic acid metabolism is important to antioxidation and detoxification rather than pathogenicity in *Magnaporthe oryzae*

**DOI:** 10.1080/21505594.2024.2444690

**Published:** 2025-01-15

**Authors:** Chang Liu, Yi Wei, Yuejia Dang, Wajjiha Batool, Xiaoning Fan, Yan Hu, Zhengquan He, Shihong Zhang

**Affiliations:** a The Key Laboratory for Extreme-Environmental Microbiology, College of Plant Protection, Shenyang Agricultural University, Shenyang, China; b College of Life and Health, Dalian University, Dalian, China; c Key Laboratory of Three Gorges Regional Plant Genetics and Germplasm Enhancement (CTGU)/Biotechnology Research Center, Three Gorges University, Yichang, China

**Keywords:** Oxalate decarboxylase (MoOxdC), *Magnaporthe oryzae*, D-erythroascorbic acid, energy metabolism, pathogenicity

## Abstract

Oxalic acid (OA), an essential pathogenic factor, has been identified in several plant pathogens, and researchers are currently pursuing studies on interference with OA metabolism as a treatment for related diseases. However, the metabolic route in *Magnaporthe oryzae* remains unknown. In this study, we describe D-erythroascorbic acid-mediated OA synthesis and its metabolic and clearance pathways in rice blast fungus. By knocking out the D-arabino-1,4-lactone oxidase gene (*Moalo1*), one-third of oxalic acid remained in *M. oryzae*, indicating a main pathway for oxalic acid production. *M. oryzae* OxdC (MoOxdC) is an oxalate decarboxylase that appears to play a role in relieving oxalic acid toxicity. Loss of *Mooxdc* does not affect mycelial growth, conidiophore development, or appressorium formation in *M. oryzae*; however, the antioxidant and pathogenic abilities of the mutant were enhanced. This is owing to *Mooxdc* deletion upregulated a series of OA metabolic genes, including the oxalate oxidase gene (*Mooxo*) and *Moalo1*, as well as both OA transporter genes. Simultaneously, as feedback to the tricarboxylic acid (TCA) cycle, the decrease of formic acid in Δ*Mooxdc* leads to the reduction of acetyl-CoA content, and two genes involved in the β-oxidation of fatty acids were also upregulated, which enhanced the fatty acid metabolism of the Δ*Mooxdc*. Overall, this work reveals the role of OA in *M. oryzae*. We found that OA metabolism was mainly involved in the growth and development of *M. oryzae*, OA as a byproduct of D-erythroascorbic acid after removing H_2_O_2_, the OA-associated pathway ensures the TCA process and ATP supply.

## Introduction

**Figure 1.**
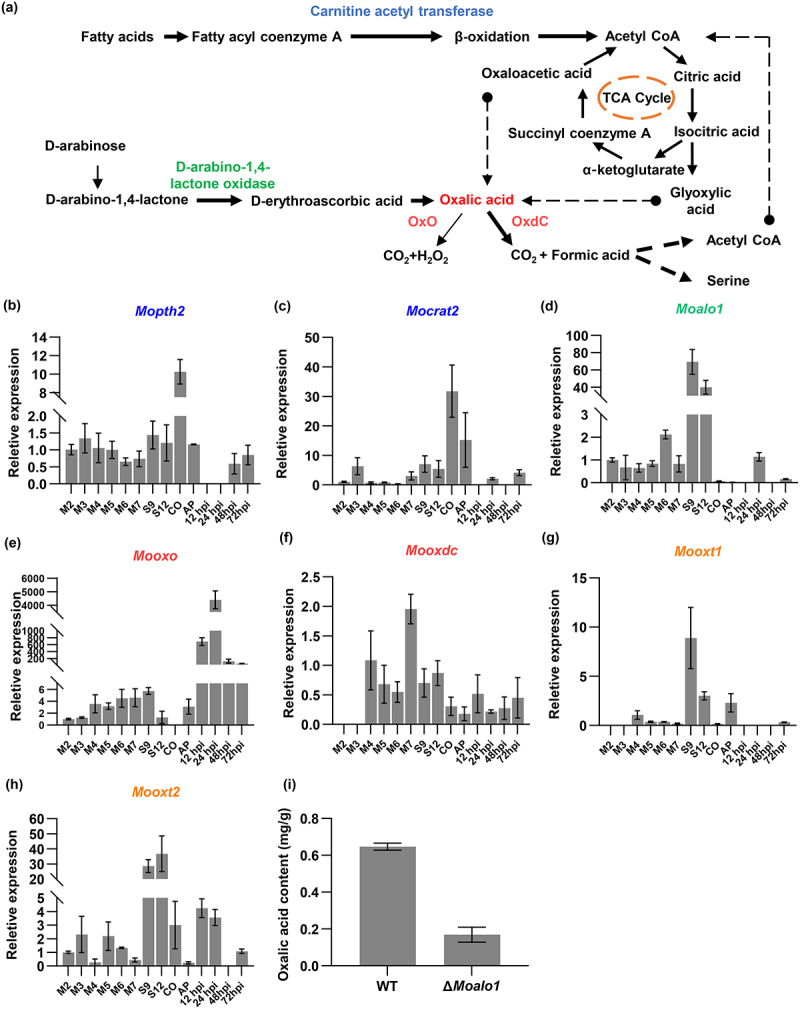
Oxalic acid synthesis, metabolism and transport in *M. oryzae*. (a) Oxalic acid synthesis, metabolism and transport pathways in fungi. OxO: oxalate oxidase; OxdC: oxalate decarboxylase (b–c) Expression dynamics of carnitine acetyltransferase in *M. oryzae*. *Mopth2* (MGG_01721), *MoCrat2* (MGG_06981). M: mycelium; S: Stalk; CO: Conidia; AP: appressorium. (d) Expression dynamics of D-arabino-1,4-lactone oxidase in *M. oryzae Moalo1* (MGG_02689). (e) Expression dynamics of oxalate oxidase in *M. oryzae Mooxo* (MGG_10252). (f) Oxalate decarboxylase expression dynamics in *M. oryzae Mooxdc* (MGG_14061). (g-h) Expression dynamics of oxalate transport proteins *M. oryzae Mooxt1* (MGG_09838); *Mooxt2* (MGG_15133). (i) Oxalic acid content in the wild-type and Δ*Moalo1*.

Oxalic acid (OA) is considered to be the virulence factor of many pathogenic fungi and is also considered to be an important intermediate metabolite that plays an important role in the growth, development and pathogenicity of fungi [1]. Pathogenic fungi exert direct toxic effects on the host by secreting oxalic acid [2]. The low pH environment caused by oxalic acid is conducive to the secretion of pathogenic fungi cell wall-degrading enzymes, providing favorable conditions for pathogenic fungi to infect plants, which are then reused by pathogens or eventually precipitate as calcium oxalate crystals [3]. In fungi, the oxalic acid synthesis pathway is very complex, and related studies have shown that oxalic acid in fungi originates from the tricarboxylic acid (TCA) cycle, D-erythroascorbic acid, and glyoxylic cycle pathways (Figure 1a) [4–7]. Oxalic acid is degraded in fungi mainly via two pathways: oxidation and decarboxylation (Figure 1a) [8]. The oxidation reaction is that oxalic acid is catalyzed by oxalate oxidase (OxO) to generate CO_2_ and H_2_O_2_. Decarboxylation is the direct decomposition of oxalic acid into formic acid and CO_2_ by oxalate decarboxylase (OxdC) [9]. Formic acid is one of the simplest organic substances that provides a source of carbon and energy for living cells [10]. Formic acid in microorganisms through assimilation, through a multi-step catalytic reaction, and finally produces acetyl-CoA, pyruvate, and serine, then enters metabolic pathways, such as the TCA cycle and glycolysis, which support microbial growth [11]. The synthesis and degradation pathways of oxalic acid ensure the circulation of substances within the cell.

Oxalate decarboxylase is a member of the Cupin protein superfamily and belongs to the bicupin subclass because it contains two Mn^2+^ binding cupin motifs [8]. Functional oxalate decarboxylase is a hexamer consisting of two trimers of the bicupin subunit [12]. OxdC is widely used in agriculture, industry, and biological detection because of its ability to directly degrade oxalic acid [8]. This enzyme has been identified and functionally analyzed in a variety of microorganisms. In bacteria, recombinant *Bacillus subtilis* oxalate decarboxylases Yvrk and YoaN possess enzymatic activity in the presence of manganese ions and oxygen [13]. Oxalate decarboxylase was discovered in the entomopathogenic nematode symbiotic *Photorhabdus luminescens* and its crystal structure was analyzed [12]. In fungi, The oxalate decarboxylase gene *Cmoxdc1* in *Coniothyrium minitans* positively regulates its tolerance to oxalic acid and ability to parasitize *Sclerotinia sclerotiorum* [14]. In fungal pathogens, oxalate decarboxylase is involved in metabolism, growth and development, pathogenicity, and so on. Oxalate decarboxylase activity was detected in the lignin-degrading fungus white rot, and the enzyme activity was increased under the induction of oxalate, which is involved in the degradation of oxalic acid at the transcriptional level [4,15]. In the white-rot fungus *Dichomitus squalens*, oxalate decarboxylase is involved in fungal primary, but not secondary, metabolism [15]. The oxalate decarboxylase gene was identified in two strains of *S. sclerotiorum* (virulent/hypovirulent), and the production of the enzyme was induced by the composition and pH of the medium [16]. The oxalate decarboxylase gene*s Ss-odc1* and *Ss-odc2* of *S. sclerotiorum* play important roles in appressorium development and virulence [17]. The content of oxalate decarboxylase was higher in *Aspergillus Niger* strains that produced a lot of citric acid [18]. Exogenous expression of oxalate decarboxylase in crops is an important strategy to reduce oxalate levels and increase tolerance to fungal pathogens. Oxalate decarboxylase is currently used to produce transgenic oxalate-tolerant plants [19]. The oxalate decarboxylase gene from *Flammulina velutipes* was transferred to soybean, which improved soybean resistance to *S. sclerotiorum* [20]. Transgenic tomatoes have reduced oxalic acid content in transgenic tomatoes and improved fruit quality [21]. In transgenic tobacco, resistance to *Fusarium oxysporum* was improved, indicating the potential of this gene to resist Fusarium wilt [22]. *Pandoraea* sp. OXJ-11 can produce oxalate decarboxylase under the induction of oxalic acid and inhibit the occurrence of *S. sclerotiorum* [23]. Expression of oxalate decarboxylase protein of *Bacillus subtilis* in rice can enhance rice blast and sheath blight resistance [24]. Oxalate decarboxylase has a great potential for medical applications. An oxalate decarboxylase gene was cloned from *Bacillus subtilis* and recombinantly expressed in plant Lactobacillus, which improved its ability to degrade oxalic acid in vitro and has applications in the treatment of kidney stones [25,26]. The *Bacillus* Yvrk gene was cloned using the mammalian expression vector pcDNA and transfected into human embryonic kidney 293 (HEK293) cells. The results showed that HEK293 cells expressing OxdC, which can degrade oxalate, can protect cells from oxidative damage; therefore, it can be used as a treatment option to prevent CaOx stone disease [27].

Mitochondria are the power plants of the cell, and the engine-TCA cycle in mitochondria is the hub of energy metabolism, converting organic materials such as sugars, lipids, and proteins into energy in the form of triphosadenine (ATP) [28]. In addition to generating energy, the TCA cycle can also participate in many biological reactions of cells by releasing reactive oxygen species (ROS) as signaling molecules, but too high ROS levels often lead to mitochondrial damage and slow TCA rates [6,29,30]. The ascorbic pathway reduces and controls ROS byproducts associated with ATP synthesis in the mitochondria [31]. As is well known, L-ascorbic acid (ASC) is an important antioxidant in plants and animals cells and plays a significant role in growth and development and defense mechanisms [32,33]. D-erythroascorbic acid (EASC) in fungi has high physicochemical and structural similarities to ascorbic acid and is a substitute for ascorbic acid. Indeed, EASC has also been shown to act as an oxidative scavenger, regulating the balance of ROS levels *in vivo* [34]. Huh et al. found that EASC is an important antioxidant component in *Saccharomyces cerevisiae* and *Candida albicans*, and that EASC is one of the virulence factors of *C.albicans* [35,36]. EASC also function in the growth and pathogenicity of *M. oryzae*. After deletion of the D-arabinono-1,4-lactone oxidase gene (*Moalo1*), which catalyzes the synthesis of EASC, the development of the appressorium of Δ*Moalo1* was abnormal, and the antioxidant ability, pathogenicity, and infection ability were decreased [37].

*S. sclerotiorum* is a type of oxalic acid-producing fungus. As is well known, oxalic acid is an important pathogenic factor for necrotrophic fungi such as *S. sclerotiorum* [2]. *S. sclerotiorum* acidifies the infected environment by secreting oxalic acid, not only creating a suitable growth environment for itself, but also causing toxic effects on host cells [38]. Excessive environmental pH can also hinder the growth and secretion of some cell wall-degrading enzymes. At this time, *S. sclerotiorum* degrades oxalic acid by secreting oxalate decarboxylase to increase its pH value [39]. The ability of *S. sclerotiorum* to respond to and regulate environmental pH is both a factor in the successful infection of the host and a self-protection mechanism for itself [40].

Rice blast is a major disease in rice production worldwide and caused by the *Magnaporthe oryzae*. Because the rice blast fungus has a complete infection cycle and multiple physiological races, it can cause large-scale diseases [41]. Related studies have shown that *M. oryzae* can also secrete oxalic acid [42]. Unlike the necrotrophic pathogen, *M. oryzae*, as a hemibiotroph pathogen, oxalic acid has not been reported as the main pathogenic factor in *M. oryzae*. And, the exact role of oxalic acid in *M. oryzae* remains unclear. Moreover, although there are many reports on oxalate decarboxylase, they mainly focus on the physiological and biochemical characteristics of the enzyme and its applications in agriculture, medicine and industry. In pathogenic fungi, there is only research on the identification of oxalate decarboxylase and the factors affecting enzyme activity. Only in *S. sclerotiorum* has it been found that the knockout of oxalate decarboxylase *Ss-odc2* leads to the development of sclerotia and toxicity defects, but its mechanism has not been elucidated [17]. We aimed to explore the main processes in which oxalic acid participates in the growth, development and pathogenicity of *M. oryzae*, and to clarify the mechanism of oxalate decarboxylase involvement in *M. oryzae* based on the acquisition of MoOxdC knockout mutant. In this study, we revealed the role of OA in *M. oryzae* as a byproduct of D-erythroascorbic acid after removing H_2_O_2_, OA associated pathway ensures the TCA process and ATP supply.

## Results

### The synthesis, metabolism and transport of oxalic acid in *M. oryzae* and closely with its growth and development

Although there have been many studies on the role of oxalic acid in oxalic acid-producing fungi, its role of oxalic acid in *M. oryzae* is still unclear. To investigate the role of oxalic acid in *M. oryzae*, we selected genes related to oxalic acid synthesis, metabolism, and transport pathways, including *Moalo1*, *Mopth2*, *Mocrat2*, *Mooxo*, *Mooxdc*, *Mooxt1* and *Mooxt2*, and analyzed their expression levels of these genes at different stages of growth and infection in *M. oryzae*. The results showed that the D-erythroascorbic acid synthesis pathway gene *Moalo1*, acetyl CoA synthesis pathway genes *Mopth2* and *Mocrat2*, oxalate decarboxylase *Mooxdc*, the oxalate transporter protein *Mooxt1* were all expressed during the growth and development stages (Figure 1b-d, 1-g). Although oxalate oxidase levels increased significantly during the infection and growth stages, the overall expression level of oxalate oxidase in *M. oryzae* is very low (Figure 1e). The expression level of another oxalate transporter, *Mooxt2* is high during the growth period and the early infection stage (Figure 1h). Previous studies have shown that the *Moalo1* gene is localized within mitochondria, and its knockout reduces the antioxidant and pathogenic abilities of *M. oryzae*. We obtained *Moalo1* knockout mutants and detected changes in the oxalic acid content in Δ*Moalo1* (Fig. S1). The results showed that the oxalic acid content in Δ*Moalo1* decreased by two-thirds compared to that in the wild type (Figure 1i). Based on the above results, we concluded that some of the genes involved in the oxalate synthesis pathway are expressed during the growth and development stages, indicating that oxalic acid is mainly involved in the growth and development of *M. oryzae*. Although previous studies have shown that the introduction of oxalate decarboxylase from *Bacillus subtilis* in rice enhances the resistance of rice to *M. oryzae*, but oxalate is not the main pathogenic factor of *M. oryzae* [24]. Moreover, in the Δ*Moalo1* mutants, the oxalic acid content decreased by two-thirds, indicating that the d-erythroascorbic acid pathway is the main pathway for oxalic acid synthesis.

### *M. oryzae* OxdC (MGG_14061) is an oxalate decarboxylase

**Figure 2.**
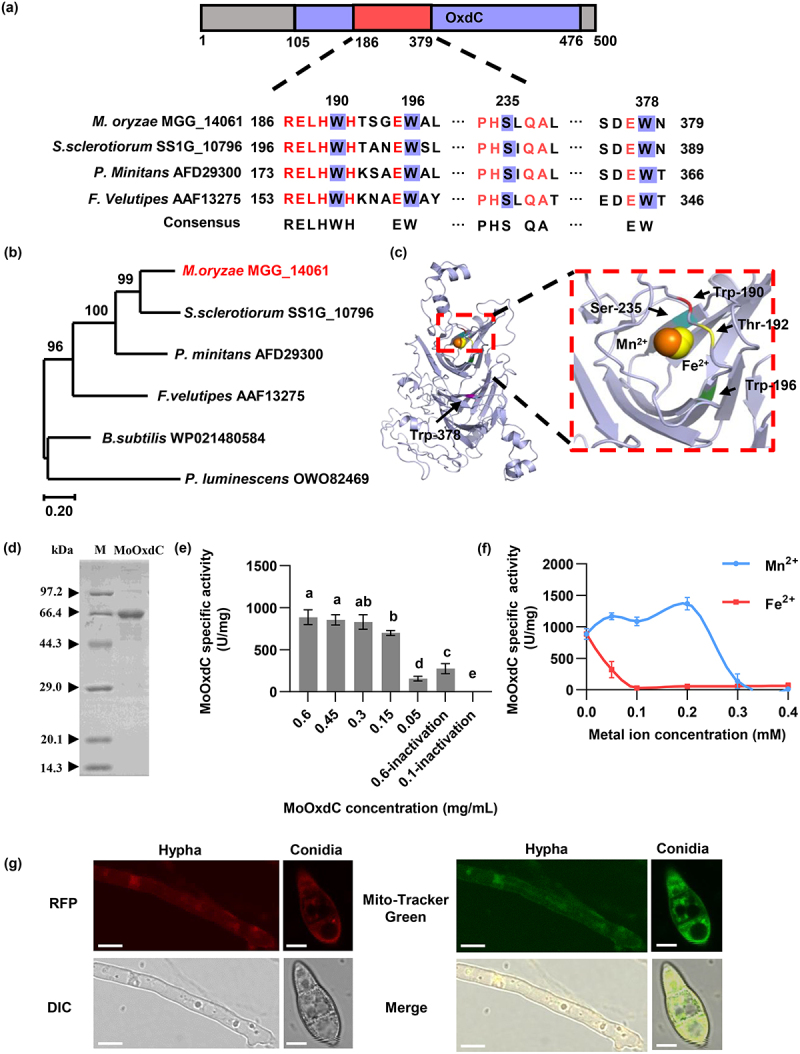
*M. oryzae* OxdC (MGG_14061) is an oxalate decarboxylase. (a) MoOxdC protein conserved domain predicted and oxalate decarboxylase protein sequence alignment in different species. (b) Phylogenetic tree of the MoOxdC protein. *M. Oryzae* (MGG_14061), *S. sclerotiorum* Ss-odc2 (SS1G_10796), *S. sclerotiorum* Ss-odc-1 (SS1G_08814), *P. minitans* (AFD29300), *F. velutipes* (AAF13275), *B. subtilis* (WP021480584), *P. luminescens* (OWO82469). (c) 3D structure and ligand binding sites and enzyme active site prediction of MoOxdC. (d) SDS-page analysis of MoOxdC after purification. M: protein marker. (e) MoOxdCOxdC enzymatic activity. Small letters indicate significant differences. difference, *p* < 0.05. (f) MoOxdC enzyme activity after addition of Mn^2+^ and Fe^2+^. (g) Observation of subcellular localization of MoOxdC. Bar = 5 μm.

To identify the function of the MGG_14061, the conserved domain of this protein was predicted using the NCBI conserved domain search service tool (https://www.ncbi.nlm.nih.gov/Structure/cdd/wrpsb.cgi). The results showed that the protein encodes a protein of 500 amino acids. The protein sequence alignments delineated an oxalate decarboxylase OxdC domain between 105–476^th^ amino acid (Figure 2a). Therefore, we rename the protein MoOxdC. We used the NCBI database to compare oxalate decarboxylase proteins in other species. Then MEGA X software was used to compare the homology of these proteins and construct the phylogenetic. According to the alignment results, the arginine 190, 196, 235, and 378 sites were conserved in other species of oxalate decarboxylase genes in the phylogenetic tree, including *S. sclerotiorum, P. minitans, F. velutipes, B. subtilis* and *P. luminescens* (Figure 2a,b). The relationship between *M. oryzae MoOxdC* and *S. sclerotiorum Ss-odc2* was the closest, followed by that between *P. minitans Cmoxdc* (Figure 2b). According to relevant research reports, *S. sclerotiorum* oxalate decarboxylase gene *Ss-odc2* impacts its appressorium development and virulence [17]. *P. minitans* oxalate decarboxylase genes *Cmoxdc1* and *Cmoxdc2* are important for hyperparasitism of *S. sclerotiorum* and secretion of antifungal substances [14]. Oxalate decarboxylase is a manganese-containing polymerase in the bicupin superfamily. Mn(II) in oxalate decarboxylase can catalyze oxalic acid decarboxylation [43]. The catalytic process occurs at the metal ion binding site [44]. And the homologous enzymes of bicupin family can bind and catalyze with other alternative metals, such as Zn^2+^, Ni^2+^, Cu^2+^ and Fe^2+^, etc [45]. In order to explore whether MoOxdC contains some metal ion binding sites and enzyme active site. Then, we used the I-TASSER server for protein structure and function prediction (zhanggroup.org) to predict the 3D model of MoOxdC, which has a Mn^2+^ and Fe^2+^ ligand, and the binding sites were located at arginine 190 (Arg-190), threonine 182 (Thr-182), arginine 196 (Arg-196), and serine 235 (Ser-235). An enzyme active site at arginine 378 (Arg-378) was also predicted (Figure 2c). According to the predicted results, Mn^2+^ and Fe^2+^ may play a role in catalyzing the enzyme activity of MoOxdC.

Oxalate decarboxylase can degrade oxalate. To identify the activity of MoOxdC, we constructed a prokaryotic expression strain of *MoOxdC* and obtained a purified protein with a size of 62 kDa (Figure 2d) and the concentration of the protein was 0.15 mg/mL. The oxalate decarboxylase activity of the purified recombinant MoOxdC protein was analyzed. MoOxdC enzyme activity increased with increasing MoOxdC dosage (Figure 2e), indicating that MoOxdC, as an oxalate decarboxylase, participates in the degradation of oxalic acid. According to the results of enzyme activity measurement, in a reaction system, when the enzyme dosage reaches 0.45 mg/mL, the specific activity of the enzyme reaches its highest level. When the enzyme dosage is increased to 0.6 mg/mL, the specific activity of the enzyme remains unchanged. This indicated that when the protein dosage is greater than 0.45 mg/mL, the maximum catalytic efficiency of MoOxdC is achieved (Figure 2e). Then, different concentrations of Mn^2+^ and Fe^2+^ were added to the reaction solution to observe the effects of the two metal ions on MoOxdC enzyme activity. The results showed that the enzyme activity of MoOxdC can be promoted when the concentration of Mn^2+^ is 0.05–0.2 mM, and the promotion effect is enhanced with an increase in the concentration of Mn^2+^. However, when the concentration of Mn^2+^ increased to 0.3–0.4 mM, the enzyme activity was inhibited, and the inhibition effect increased with the increase in Mn^2+^ concentration, and the enzyme activity was completely inhibited when the concentration was 0.4 mM (Figure 2f). In contrast to Mn^2+^, Fe^2+^ inhibited enzyme activity when its concentration was 0.05–0.4 mM completely (Figure 2f). The above results showed that MoOxdC acted as oxalate decarboxylase and had the highest promoting effect on enzyme activity when Mn^2+^ was 0.2 mM, while Fe^2+^ had an inhibitory effect on enzyme activity.

To determine the function of MoOxdC in *M. oryzae*, we used the homologous recombination targeted gene deletion technique and successfully generated *Mooxdc* deletion strains (Δ*Mooxdc-85*, Δ*Mooxdc-106*) (Fig. S2a-c). The transformants were screened by PCR using the primers *MoOxdC*-A-HYG (UAH), HYG- *MoOxdC*-B (UBH), MGG_15997 (CONF), and *MoOxdC* (ORF). After verification, the two transformants were identified as *MoOxdC* deletion strains (Fig. S2b), and the qPCR data indicated that the relative expression of *MoOxdC* in the mutants was downregulated to zero, indicating that we successfully knocked out the gene (Fig. S2c). We then used the vector pKD7-Red to construct a recombinant vector, pKD7-Red-MoOxdC, which was transformed into the deletion mutants (Fig. S3). Firstly, we determined MoOxdC’s subcellular localization. We predicted the subcellular location of MoOxdC using ProtComp 9.0, and the results showed that MoOxdC was located in the mitochondria (Table S1). The subcellular localization of MoOxdC in mycelia and conidia was observed by laser confocal microscopy. The mycelia and conidia of Δ*Mooxdc/oxdc* were stained with MitoTracker Green mitochondrial fluorescent dye. The results showed that the green fluorescence of MoOxdC-RFP overlapped with that of mitochondria stained with MitoTracker Green (Figure 2g). These results indicate that MoOxdC is localized to the mitochondria of *M. oryzae*.

### MoOxdC deletion strains fail to degrade oxalic acid

**Figure 3.**
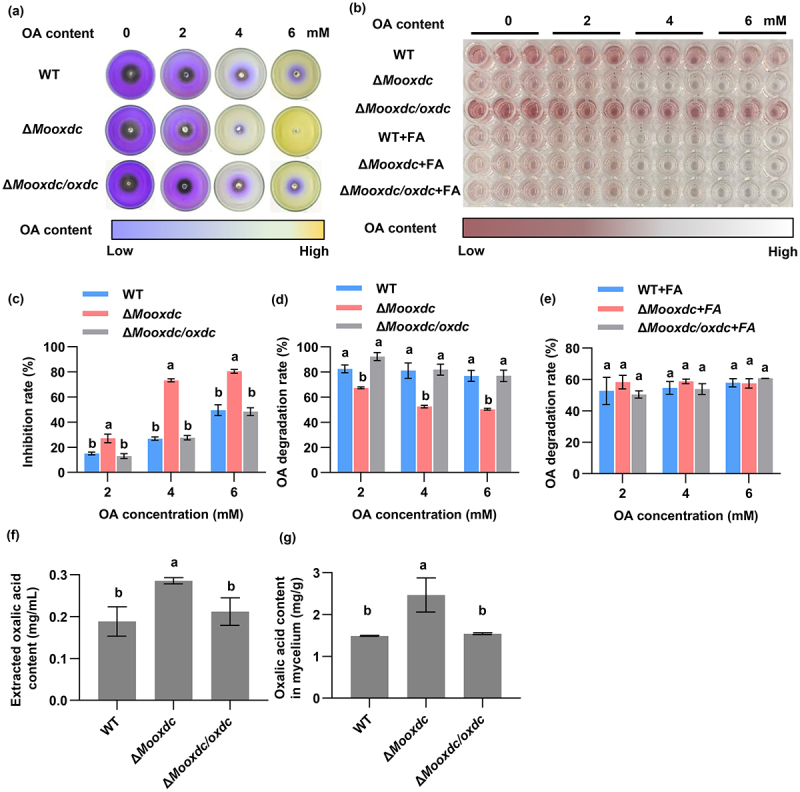
Tolerance of the mutants and the wild type of *M. oryzae* to oxalic acid (OA). (a) Growth status of strains on PDA which contain different concentrations OA for 7 days. (b) OA content in PDB or PDB added 1 mM formic acid containing different concentrations OA which cultured Δ*Mooxdc*, wild type and complementary strain for 7 days. (c) Inhibition rate of strains on PDA which contain different concentration OA. (d) OA degradation rate of the Δ*Mooxdc*, wild type and complementary strains after 7 days cultured on the PDB containing 1 mM formic acid and different concentration OA. (e) OA degradation rate of the Δ*Mooxdc*, wild type and complementary strains after 7 days cultured on the PDB containing different concentration OA. (f) Determination of OA secretion by Δ*Mooxdc*, wild type and complementary strains. (g) Determination of OA content in mycelia of Δ*Mooxdc*, wild type and complementary strains. Small letter represent significant difference, *p* < 0.05.

Based on the characteristics of oxalate decarboxylase. We tested the effect of Δ*Mooxdc* on oxalic acid secretion and degradation in *M. oryzae*. Wild-type, mutant, and complementary strains were cultured on PDA plates containing different concentrations of oxalic acid, bromophenol blue was added as an indicator, pH values from acid to base, and medium colors ranging from yellow to blue. Through the solid plate culture, the oxalic acid concentration range of 0–6 mM, around the colony turned blue instead of yellow, indicating that oxalic acid could be degraded (Figure 3a). Compared to the wild-type and complementary strains, the growth of Δ*Mooxdc* was significantly inhibited when the concentration of OA was 2, 4, and 6 mM (Figure 3a, c). Therefore, *MoOxdC* regulates the ability of *M. oryzae* to degrade OA. At the same time, we measured the oxalic acid content in the potato dextrose broth (PDB) medium after 7 days of culture, which contained different concentrations of oxalic acid. When the concentration of oxalic acid was 2, 4, and 6 mM, the oxalic acid content in the culture solution of mutants was higher than that in the wild-type and complementary strains (Figure 3b). We also found that PDB without oxalic acid showed that *M. oryzae* can secrete oxalic acid. Moreover, the oxalic acid content secreted by the Δ*Mooxdc* mutants was higher than that in the wild type (Figure 3b,f). The oxalic acid degradation rate result showed that when the concentration of oxalic acid was 2, 4 and 6 mM, the degradation rate of Δ*Mooxdc* was lower than that of wild-type and complementary strains (Figure 3d). Based on the principle of feedback adjustment, we added 1 mM formic acid to the PDB medium containing different concentrations of oxalic acid. The results showed that the ability of the wild-type and complementary strains to degrade oxalic acid was similar to that of the mutant strain after adding formic acid (Figure 3b,e). These results indicate that *MoOxdC* has oxalate decarboxylase activity in *M. oryzae*, and deletion of *MoOxdC* reduces the tolerance and degradation ability of *M. oryzae* to oxalic acid (OA).

### MoOxdC deletion strains exhibit stronger antioxidant abilities

We performed a growth assay to analyze the role of *MoOxdC* in *M. oryzae* vegetative development of *M. oryzae*. When cultured on CM and PDA medium for 6 days, there was no significant difference in the growth rate between Δ*Mooxdc* and the wild type (Fig. S4). Thus, we hypothesized that *MoOxdC* is involved in conidial formation, stress resistance, and pathogenicity of *M. oryzae*. We examined the development of conidia and appressorium in *M. oryzae*. The conidiation and conidiospore numbers also showed no difference (Fig. S5). Conidia germination and appressorium formation were observed, and the germination rate and appressorium formation rate of Δ*Mooxdc* and the wild type were the same (Fig. S6).

**Figure 4.**
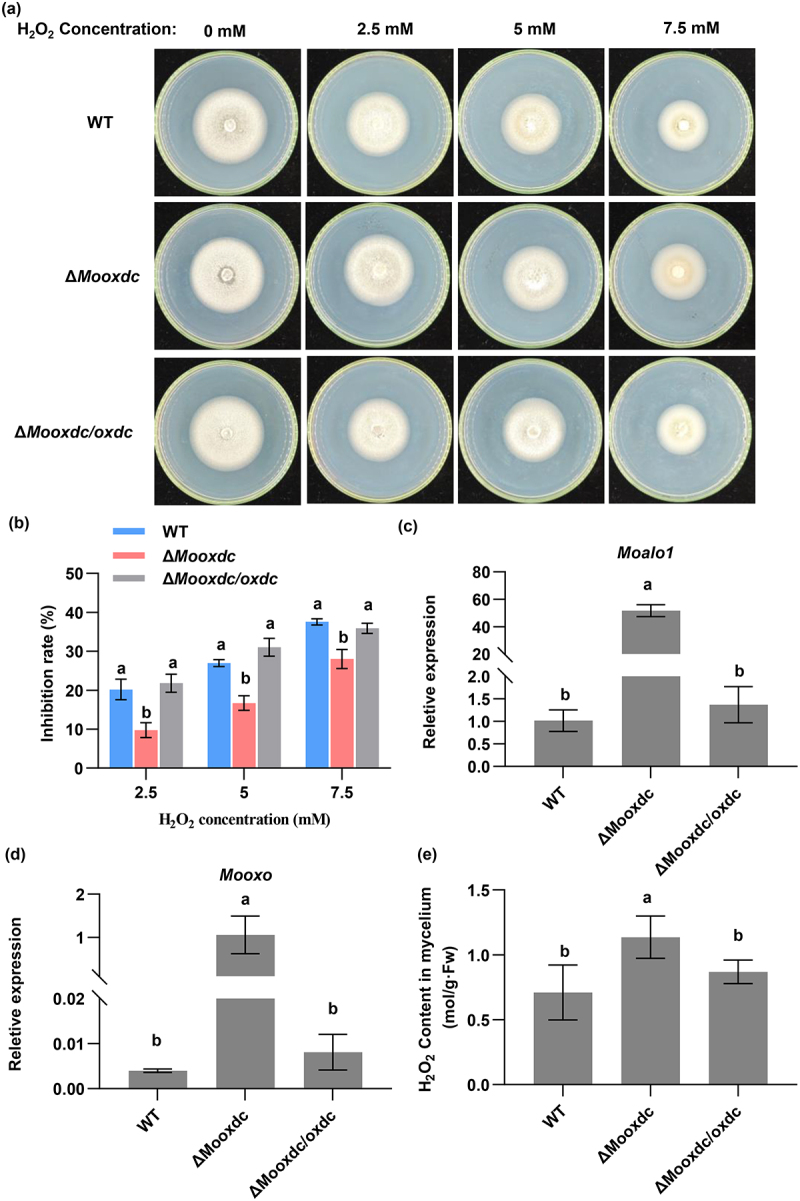
MoOxdC deletion strains exhibit stronger antioxidant abilities. (a) Δ*Mooxdc*, wild type and complementary strain growth on complete agar medium under H_2_O_2_ stress. (b) Statistical analysis of mycelial growth of Δ*Mooxdc*, wild type and complementary strain on complete agar medium under H_2_O_2_ stress. (c) Expression analysis of *Moalo1* (MGG_02689) gene by quantitative real-time polymerase chain reaction (qRT-PCR) in Δ*Mooxdc*, wild type and complementary strain. (d) Expression analysis of *MoOxO* (MGG_10252) gene by quantitative real-time polymerase chain reaction (qRT-PCR) in Δ*Mooxdc*, wild type and complementary strain. (e) Determination of H_2_O_2_ content in Δ*Mooxdc*, wild type and complementary strain mycelial.

We tested the antioxidant ability of Δ*Mooxdc*. The H_2_O_2_ stress results showed that the wild-type and complementary strains were more sensitive to exogenous H_2_O_2_ than the Δ*Mooxdc* mutants under the H_2_O_2_ stress (Figure 4a,b). Fungi produce ascorbic acid analogue EASC through D-arabino-1,4-lactone oxidase (ALO1), which participates in the synthesis of oxalic acid and acts as an antioxidant in the clearance of ROS [7]. So we speculate that the deletion of MoOxdC affects the EASC pathway and the oxidative stress ability of *M. oryzae*, thereby affecting the tolerance of *M. oryzae* to H_2_O_2_. As previous studies have shown that the ALO1 gene catalyzes the final step of EASC synthesis [35,46]. Therefore, we analyzed the expression of a gene in this pathway in mutants and the wild type, which encodes D-arabino-1,4-lactone oxidase (*Moalo1*). EASC production was characterized by the expression level of *Moalo1*. The expression of this gene was significantly upregulated in Δ*Mooxdc* compared to that in the wild-type and complementary strains (Figure 4c). As reported earlier, the oxidative stress ability of Δ*Moalo1* decreased, which is consistent with our results [36].

We hypothesized that oxalic acid degradation is completed by another oxalic acid-degrading enzyme, oxalate oxidase, to eliminate the damage caused by oxalic acid accumulation in cells. By comparing the genome of *M. oryzae*, there is an oxalate oxidase *MoOxO*, we detected the expression of this oxalate oxidase in the wild type, Δ*Mooxdc/oxdc* and Δ*Mooxdc*. According to the results, the expression of *MoOxO* was significantly increased when *MoOxdC* was knocked out (Figure 4d). With an increase in *MoOxO* expression, the H_2_O_2_ content in Δ*Mooxdc* also increased (Figure 4e). To keep H_2_O_2_ in the cell stable, excessive H_2_O_2_ induced the expression of the *Moalo1* gene, accelerated the EASC pathway, and increased the antioxidant capacity of *M. oryzae*. According to the above results, MoOxdC had little correlation with the vegetative growth of *M. oryzae*. After deleting *MoOxdC*, the oxalic acid degradation pathway of oxalate oxidase was activated, and the degradation product, H_2_O_2_ induced the production of EASC, which enhanced the resistance of Δ*Mooxdc* to H_2_O_2_.

### MoOxdC deletion strains exhibit stronger pathogenic abilities

**Figure 5.**
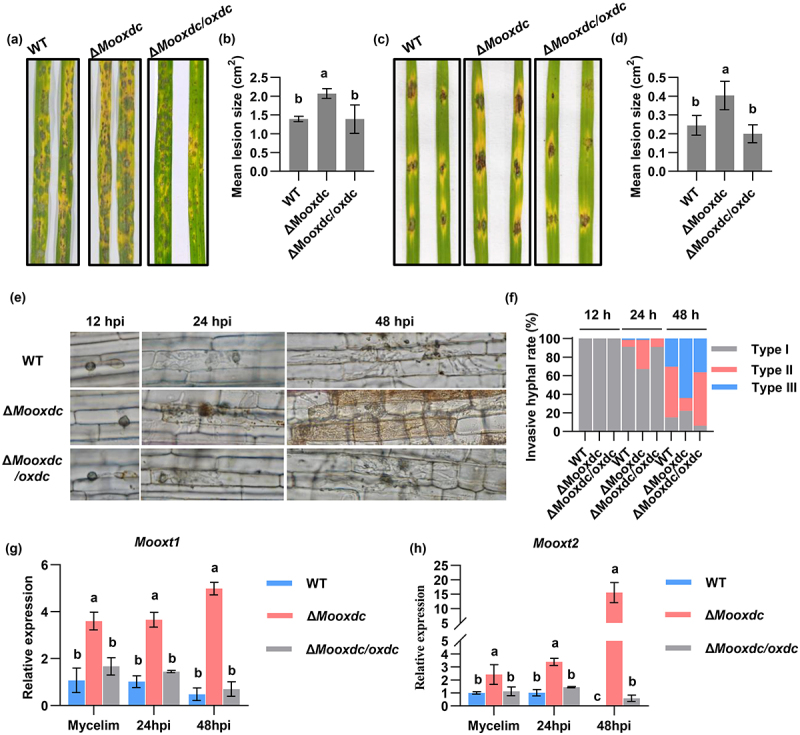
MoOxdC deletion strains exhibit stronger pathogenic abilities. (a) Δ*Mooxdc*, wild type and complementary strain spraying pathogenicity test on rice. (b) Mean lesion size of spraying pathogenicity rice leaves. (c) Δ*Mooxdc*, wild type and complementary strain point pathogenicity test on rice. (d) Mean lesion size of point pathogenicity rice leaves. (e) Microscopic observation of penetration and infectious hyphae expansion on rice leaf-sheath cells. (f) Infectious rate of Δ*Mooxdc*, wild type and complementary strain. (g-h) expression analysis of *Mooxt1* (MGG_09838) gene and *Mooxt2* (MGG_15133) gene by quantitative real-time polymerase chain reaction (qRT-PCR) in Δ*Mooxdc*, wild type and complementary strain between mycelial and infection stage.

**Figure 6.**
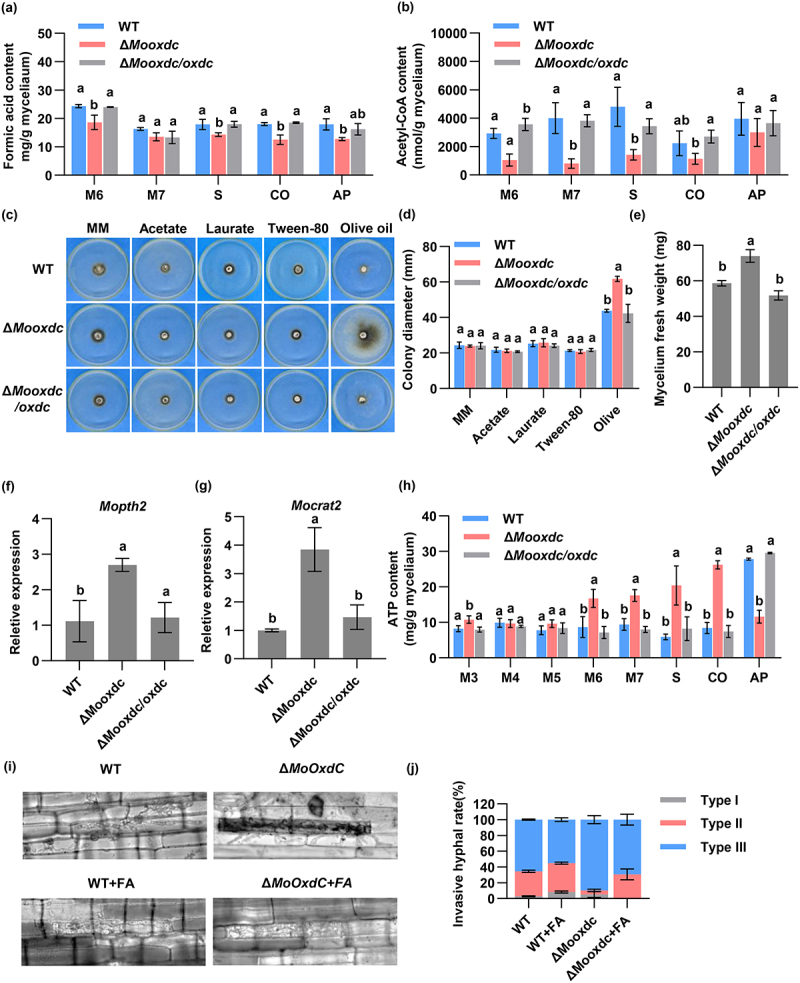
Deletion of MoOxdC resulting in enhanced fatty acid metabolism. (a) Formic acid content in Δ*Mooxdc*, wild type and complementary strain at the different growth stages. (b) Acetyl-CoA content in Δ*Mooxdc*, wild type and complementary strain at the different growth stages. M: mycelium, S: Stalk; CO: Conidia; AP: appressorium. (c) The Δ*Mooxdc*, wild type and complementary strain were inoculated onto MM plates or MM with different fatty acids as carbon sources, cultured at 28°C for 14 days, and photographed. (d) Statistical analysis of the colony diameter of the different strains on different media. (e) Statistical analysis of the mycelium fresh weight of the different strains on olive oil medium. (f) The expression of *Mopth2* (MGG_01721) in Δ*Mooxdc*, wild type and complementary strain. (g) The expression of *Mocrat2* (MGG_06981) in Δ*Mooxdc*, wild type and complementary strain. (h) The content of ATP in the Δ*Mooxdc*, wild type and complementary strain. (i) Microscopic observation of penetration and infectious hyphae expansion on rice leaf-sheath cells after inoculated with the conidial suspension add and without FA. FA: formic acid; concentration of FA is 1 mM. (j) Invasive hyphal rate.

To further explore the function of MoOxdC in *M. oryzae*, rice leaves were inoculated with a conidia suspension containing 5 × 10^4^ conidia/mL using spraying and point inoculation methods, respectively. In the progress of pathogenicity, we found that the formation of lesions on the leaves of the mutant-treated plants was faster than that of the wild-type and complementary strains post-inoculation (hpi). After 5 days, the mutant strains caused larger lesion areas than the wild-type and complementary strains (Figure 5a–d). The leaf sheath was infected with conidia for microscopic observation. The infection degree of *M. oryzae* to rice leaf sheath was divided into three types, type І is the formation of appressorium, type ІІ is the infection of the first cell, and the type ІІІ is the infection of second and more cells. The infection level of the leaf sheath was determined according to this standard. The results showed that there was no significant difference at 12 hours after infection (Figure 5e,f). At 24 hpi, 8.9 % of wild type, 32 % of Δ*Mooxdc* and 9.1 % of Δ*Mooxdc/oxdc* infects in the first cell. At 48 hpi, 30.35 % of wild type, 63.8 % of Δ*Mooxdc* and 36.3 % of Δ*Mooxdc/oxdc* infects in the second cell (Figure 6f). These results indicate that the infection rate of Δ*Mooxdc* is faster than that of the wild type, *MoOxdC* is involved in the infection process of *M. oryzae*.

According to the above findings, the oxalic acid content in the mycelium and secreted oxalic acid of Δ*Mooxdc* was higher than that of the wild type (Figure 3f–g). This is due to the absence of *MoOxdC*, which leads to a reduction in the ability of *M. oryzae* to degrade oxalic acid; thus, the oxalic acid content in cells increases. We found that the expression level of *Mooxo* was extremely low in the wild type (Figure 4d). Although the expression level of *Mooxo* was significantly upregulated after MoOxdC knockout, the expression level of *Mooxo* was also very low compared to other genes. Therefore, we believe that oxalate oxidase can reduces oxalic acid accumulation, but oxalate oxidase can only partially reduce the excess oxalic acid. Related studies have shown that the quantity of oxalic acid transporter FpOAR in brown rot fungus *Fomitopsis palustris* is related to the oxalic acid in the culture medium and is responsible for the secretion of oxalic acid [47]. Regulation of oxalate excretion in the intestine by oxalate transporter SLC26A6 in rats [48]. And we measured the oxalic acid content in the mycelium of wild type, Δ*Mooxdc* and Δ*Mooxdc*/*oxdc*, and the results showed that the oxalic acid content in the mutant significantly increased (Figure 3g). Therefore, we infer that the increase in oxalic acid content in the Δ*Mooxdc* induces an upregulation of oxalic acid transporter expression, which in turn leads to an increase in the outward secretion of oxalic acid. To further verify this result, we predicted two oxalic acid transporters, *Mooxt1* and *Mooxt2* (MGG_09838, MGG_15133), in *M. oryzae* and measured their expression. The results showed that the expression of these two oxalic acid transporters in the mutants was significantly upregulated compared with the wild type between the growth and infection stages (Figure 5g,h). These results indicated that *MoOxdC* negatively regulates the pathogenicity and infectivity of *M. oryzae* in rice. The absence of *MoOxdC* leads to the upregulation of oxalic acid transport protein, leading to increased oxalic acid secretion, which is one of the reasons for increased pathogenicity.

### Deletion of MoOxdC resulting in enhanced fatty acid metabolism

Oxalate decarboxylase degrades oxalic acid to produce formic acid, which is involved in cellular synthesis of acetyl-CoA and serine [11]. Previous studies have shown that fatty acid β-oxidation biogenesis is impaired during infection, and carnitine acetyltransferase, which regulates fatty acid β-oxidation, has been found to be involved in the synthesis of oxalic acid [49]. And fatty acid can serve as one of the precursors for the synthesis of acetyl CoA, acetyl CoA participates in the TCA cycle (Figure 1a). Based on these, we speculate whether the absence of MoOxdC will affect the fatty acid metabolism and TCA cycle of *M. oryzae*. So, We first tested the concent of formic acid and acetyl CoA in wild type, Δ*Mooxdc* and Δ*Mooxdc/oxdc*. The result showed that, in the Δ*Mooxdc* mutant, the production of formic acid and acetyl CoA were significantly reduced compared to wild type and complementary strains (Figure 6a-b). According to the principle of metabolic feedback regulation, the decrease in formic acid content in the Δ*Mooxdc* mutant leads to a decrease in acetyl CoA content, which may accelerate the synthesis pathway of acetyl CoA. Then, the wild-type, Δ*Mooxdc* and complementary strains were inoculated onto minimal medium (MM) plates containing fatty acids with various chain lengths, including short-chain (acetate), medium-chain (laurate), and long-chain (olive oil) fatty acids. After 14 days of incubation, the growth rate of Δ*Mooxdc* was not significantly different from that of the wild type on acetate, laurate, and Tween-80 plates, and the growth rate of Δ*Mooxdc* was significantly higher than that of the wild type on olive oil plates (Figure 6c,d). Similarly, the biomass of the mutant grown on olive oil medium was significantly higher than that of the wild-type (Figure 6e). This indicates that MoOxdC is involved in the oxidation of long-chain fatty acids. Carnitine acetyltransferase participates in the fatty acid oxidation pathway to acetyl-coenzyme A, which then enters the TCA cycle, and oxaloacetic acid in the TCA cycle produces oxalic acid (Figure 1a). Therefore, we analyzed the expression of two carnitine acetyltransferase genes, *Mopth2* and *Mocrat2* (MGG_01721 and MGG_06981), in Δ*Mooxdc*, wild type, and complementary strains using qRT-PCR. These two genes are also up-regulated in Δ*Mooxdc* (Figure 6f,g). Carnitine acetyltransferase is mainly responsible for the transport of long-chain fatty acids, which is consistent with an increase in carnitine acetyltransferase gene expression [50]. The TCA cycle is the energy center of the organism. Due to accelerated fatty acid metabolism, we measured the ATP content in the mutants and wild type at various stages of growth and development. The results showed that the ATP content of the mutans was higher than that of the wild type from the mycelium to conidia. In the appressorium stage, the ATP content of the mutant was significantly lower than that of wild type (Figure 6h). This indicates that MoOxdC affects fatty acid metabolism in *M. oryzae*. The above results illustrate that deletion of MoOxdC leads to an increase in carnitine acetyl transferase gene expression in the oxalic acid synthesis pathway, which enhances the oxidation of fatty acids and ATP products, and thus enhances the infection ability of *M. oryzae*.

Oxalate decarboxylase degrades oxalate into formic acid and CO_2_. Modulation of action according to enzyme feedback. We added 1 mm of formic acid into the conidia solution of the wild type and Δ*Mooxdc* mutant, and then injected it into the leaf sheath to observe whether the infection of rice with different strains changed. We found that after the addition of exogenous formic acid, the infection rate of both the wild type and mutant decreased, and the infection degree of Δ*Mooxdc* recovered to be consistent with that of the wild type (Figure 6i,j). This result also shows that exogenous formic acid supplementation can normalize formic acid metabolism and restore normal growth of infected hyphae.

## Discussion

**Figure 7.**
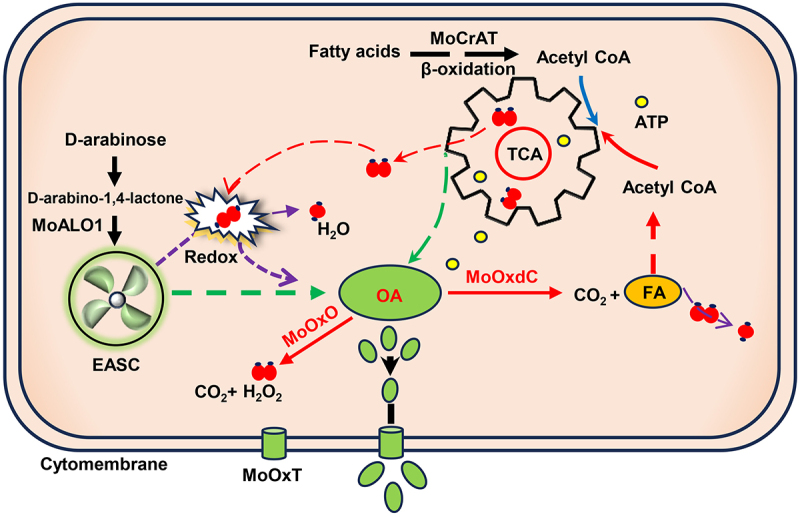
Model of oxalic acid production and removal in *M. oryzae*. In *M. oryzae*, the D-erythroascorbic acid (EASC) pathway is the main pathway for synthesizing oxalic acid. Mitochondria serve as intracellular power plants, with TCA cycle being the main energy metabolism process. During the process of power generation, “heat” (H_2_O_2_) is released. EASC is like a radiator, responsible for removing excess “heat” (H_2_O_2_) and ensuring normal cellular metabolism. And, the formation of oxalic acid is a product of EASC clearing excess H_2_O_2_ by TCA cycle. Meanwhile, oxalic acid forms formic acid, which enters the one-carbon cycle to form acetyl-CoA and then enters the TCA cycle. From the perspective of ATP formation, the formation of oxalic acid ensures the carbon cycle and thus the progress of energy metabolism. When the oxalic acid degradation pathway is disrupted (Δ*Mooxdc*), the *M. oryzae* produces a stress response, which activates the up-regulation of oxalate oxidase and oxalate transporter protein expression to reduce the damage of excessive oxalate to the cell.

Oxalic acid is widely observed as a pathogenic factor in plant pathogenic fungi [51]. Deletion of *S. sclerotiorum* oxaloacetate acetylhydrolase significantly reduced oxalic acid in *S. sclerotiorum* was significantly reduced, and its pathogenicity was also significantly reduced [52]. However, the role of oxalic acid in *M. oryzae* remains unclear. In this study, we demonstrate that the EASC pathway is the main pathway for oxalic acid synthesis in *M. oryzae*. Oxalic acid can act as a pathogenic factor in *M. oryzae* oryzae but is not the main pathogenic factor. The main function of oxalic acid in *M. oryzae* is to connect the EASC pathway and TCA cycle, reducing the damage of the TCA byproduct H_2_O_2_ to the mitochondria and ensuring the carbon cycle within the cell (Figure 7).

In fungi, oxalic acid is mainly produced through the EASC, TCA cycle, and glyoxylate cycle pathways and is degraded through oxalate decarboxylase and oxalate oxidase [53–55]. Simultaneously, oxalic acid is secreted into the cell through oxalate transporter proteins [47]. We found that most of the genes involved in the oxalic acid synthesis, degradation, and transport pathways were expressed during the growth and development stages of *M. oryzae* (Figure 1b-d, 1-h). In Δ*Moalo1*, the oxalic acid content decreased by two-thirds compared to that in the wild-type, indicating that the EASC pathway is the main pathway for oxalic acid synthesis (Figure 1i). We did not measure changes in oxalic acid content after disruption of the the glyoxylate cycle and TCA cycle pathways. Because the TCA and glyoxylate cycle pathways of oxalic acid are complex. We found that several genes related to oxalic acid synthesis in *M. oryzae* have been studied. For example, knockout of carnitine acetyltransferase upstream of the TCA cycle and glyoxylate cycle leads to abnormal development of *M. oryzae* appressorium and decreased lipid metabolism ability [56,57]. Knockout of isocitrate lyase in the glyoxylate pathway reduces the virulence and ability of *M. oryzae* to utilize fatty acids [58]. And the glyoxylate transaminase in the glyoxylate cycle also affect the lipid metabolism of *M. oryzae* [59]. However, there is currently no relevant research on the degradation pathway of oxalic acid in *M. oryzae*. So we focus on the degradation pathways of oxalic acid.

Oxalate decarboxylase (OxdC) is widely present in various fungi and has various functions [60]. OxdC in pathogenic fungi participates in growth and development, energy metabolism, and virulence [17]. OxdC genes from *F. velutipes* have been widely used in transgenic plants to improve the resistance of transgenic plants to pathogenic fungi that secrete oxalic acid as an important virulence factor, such as *S. sclerotiorum* [61], *F. oxysporum* [22], and *Moniliophthora perniciosa* [62]. OxdC genes in some biocontrol fungi, such as *Trichoderma afroharzianum* and *Coniothyrium minitans*, are involved in pathogen inhibition through oxalate degradation [15,63]. However, the function of oxalate decarboxylase in *M. oryzae* is unclear. In this study, we identified oxalate decarboxylase MoOxdC in *M. oryzae* (Figure 2). Mn^2+^ is the cofactor of *MoOxdC* enzyme activity, and Fe^2+^ is an inhibitory factor of enzyme activity (Figure 2e,f). Moreover, we predicted the ligand binding site of Mn^2+^ and Fe^2+^, and the active site of MoOxdC (Figure 2c), these predicted sites may be involved in the process of Mn^2+^/Fe^2+^ catalyzing MoOxdC activity.

Compared with the wild-type, Δ*Mooxdc* significantly decreased the ability to degrade oxalic acid (Figure 3). After *MoOxdC* deletion, oxalic acid production increased (Figures 3b,d,). However, a high oxalic acid content inhibits fungal growth [64]. To detoxify, *M. oryzae* activates another oxalate degradation pathway involving oxalate oxidase (Figure 4d). Interestingly, the expression of *Mooxo* was indeed increased in the Δ*Mooxdc* mutant and low expression of this gene in the wild type (Figure 4d). Simultaneously, the TCA cycle and oxalate oxidase degradation of oxalic acid produce more H_2_O_2_ as a negative product. ROS in fungi are involved in energy metabolism, stress resistance, and signal transduction [65], and excess ROS in cells can have toxic effects on mitochondria. According to the experimental results of H_2_O_2_ stress, the tolerance of Δ*Mooxdc* to H_2_O_2_ was significantly enhanced compared with that of the wild type (Figures 4a,b). The increase in H_2_O_2_ content induced an increase in EASC content to maintain intracellular ROS homeostasis. EASC is an antioxidant that can remove ROS [66]. We found that studies have shown that the expression of the D-arabino-1,4-lactone oxidase (*MoAlo1*) gene is involved in the antioxidant ability of *M. oryzae*, which is in the ESAC pathway of oxalic acid synthesis, and the expression of *Moalo1* in Δ*Mooxdc* was upregulated (Figure 4c). Therefore, we believe that an increase in EASC production enhances the antioxidant capacity of Δ*Mooxdc*. After the oxalic acid decarboxylation pathway was blocked, in addition to oxalate oxidase, which played a role in clearing oxalic acid in the mutant, the expression of the oxalic acid transporter gene was also upregulated to transport excess oxalic acid inside the cell to the outside of the cell (Figures 5g,h). This also indicates that the increased pathogenicity of Δ*Mooxdc* is due to its enhanced antioxidant capacity and increased oxalic acid (Figures 5a-d; Figure 4).

The whole process of plant infection of *M. oryzae* from conidial germination to infection hypha development is driven by the storage substances carried in the conidia [67]. Previous experiments have shown that lipids rapidly degraded into fatty acids and glycerol during the development of the appressorium on the leaf surface of conidia [68]. Fatty acids are key molecules for energy storage and production in cells [69]. Fatty acids produce acetyl-CoA mainly through β-oxidation, and acetyl-CoA enters different organelles through the TCA cycle and glyoxylic acid cycle to participate in the growth of fungi on different carbon sources [70]. Fatty acid metabolism and consequent acetyl-CoA are also necessary for *M. oryzae* to complete its infective developmental stage and enter the host plant [67]. Carnitine acetyltransferases play an important role in fatty acid metabolism [49]. Research has shown that the homologous proteins of carnitine acetyltransferase in *S. sclerotiorum* are also involved in the synthesis of oxalic acid [49]. In this study, our results showed that the rate of leaf sheath infection by Δ*Mooxdc* was significantly increased compared to that of the wild type (Figure 5e,f). At the same time, the expression of carnitine acetyltransferase (MGG_01721 and MGG_06981) was significantly up-regulated in the Δ*Mooxdc* mutants observed which is involved in fatty acid β-oxidation and TCA cycle processes, was significantly upregulated in the Δ*Mooxdc* mutants (Figure 6d,e). Moreover, the ability of Δ*Mooxdc* to degrade long-chain fatty acids was significantly enhanced (Figure 6a-c). Through our data and analysis, we can see that Δ*Mooxdc* hinders formic acid production, and reduced acetyl-CoA synthesis may lead to a slower TCA cycle (Figure 6a-b). However, mycelium growth requires sufficient energy, increasing the amount of enzymes associated with the upstream pathway (fatty acid β-oxidation) to ensure adequate energy supply. Therefore, the expression of the carnitine acetyltransferase gene, which regulates fatty acid oxidation, was upregulated in the Δ*Mooxdc* mutant, fatty acid metabolism of *M. oryzae* was accelerated, and infection of rice by *M. oryzae* was promoted. These results suggest that the absence of MoOxdC enhances the fatty acid metabolism process and promotes *M. oryzae* infection of *M. oryzae*.

As MoOxdC as an oxalate decarboxylase, knocking out MoOxdC leads to a decrease in the Δ*Mooxdc*’s ability to degrade oxalic acid, resulting in an increase in oxalic acid content in vivo. It is interesting that Δ*Mooxdc* also increased the secretion of oxalic acid, which is due to the upregulation of Oxalate transporter expression induced by the increase in oxalic acid content in vivo, thereby increasing the efflux of oxalic acid. The antioxidant capacity of Δ*Mooxdc* is enhanced, which is due to the degradation of excess oxalic acid in vivo by MoOxO, leading to an increase in H_2_O_2_ production, induction of upregulation of *Moalo1* gene expression, and an increase in EASC production, thereby improving Δ*Mooxdc*’s antioxidant capacity. At the same time, we also found that the content of formic acid in Δ*Mooxdc* decreased, and formic acid can generate acetyl CoA. The content of acetyl CoA in Δ*Mooxdc* also decreased. Due to metabolic feedback regulation, the expression level of the upstream genes *Mopth2* and *Mocrat2* are involved in acetyl CoA synthesis is upregulated, enhancing the fatty acid metabolism ability and ATP product of Δ*Mooxdc*. Due to the increased secretion of oxalic acid by Δ*Mooxdc*, its antioxidant capacity and lipid metabolism ability are enhanced, which is the reason for its increased infectivity and pathogenicity.

In summary, our study elucidates the role of oxalic acid in *M. oryzae*. First, oxalic acid is a product of ESAC for clearing oxidative stress. Second, from the perspective of ATP formation, oxalic acid is a by-product of the TCA cycle. Oxalic acid formation ensures the carbon cycle in vivo, allowing the TCA cycle to proceed normally. In our study, the antioxidant, pathogenic, infectious, and fatty acid metabolism enhancement of Δ*Mooxdc* was a stress response of cells to normal life activities (Figure 7).

## Experimental procedures

### Fungal strains and culture conditions

*Magnaporthe oryzae* wild-type strain JJ88 was used in this study. JJ88 was isolated and purified from *Oryza sativa* cultivar Jijing88, a variety that is widely planted in Jilin Province, China. All *M. oryzae* strains were cultured on CM, PDA, MM, and OMA medium, under 28 °C, respectively.

### MoOxdC bioinformatics analysis

Nucleotide and protein sequences were downloaded from the NCBI database (https://www.ncbi.nlm.nih.gov/). The gene Loucs_tag was MGG_14061 and the GenBank number was XM_003721447.1. Use the NCBI-Blastp (https://blast.ncbi.nlm.nih.gov/Blast.cgi) to find the homologous sequence of *MoOxdC* gene. Multi-sequence alignment of amino acid sequences was conducted using DNAMAN software, and the alignment results were used to generate a phylogenetic tree using the maximum likelihood method of MEGA X software. The three-dimensional structure of the protein was predicted using the I-TASSER server for protein structure and function prediction (zhanggroup.org). The predicted molecular weight of the protein was calculated using the online tool ExPASy (Expasy – Compute pI/Mw tool).

### Expression, purification and determination of MoOxdC activity

The full-length MoOxdC cDNA fragment was amplified and the PCR product was subcloned into the pMD-19T vector. Sequencing confirmed that the sequence was correct. The MoOxdC cDNA fragment was recovered by double digestion (EcoR І, Not І). The fragment was then ligated into the prokaryotic expression vector, pET-28a (+). *Escherichia coli* cells harboring the pET-28a-MoOxdC plasmid were transferred into BL21 (DE3) cells. Single clones of BL21 with pET-28a-MoOxdC were selected and cultured overnight at 37°C 180 rpm. The bacterial solution (2 mL) was inoculated in 200 mL of liquid LB medium when the OD reached 0.6. Then add IPTG to achieve a final concentration of 1 mM/L, culture for 3 hrs at 37°C 180 rpm. The bacterial solution was collected and centrifuged at 4 °C 12,000 rpm 3 min. And then it goes through cell destruction and recombinant protein was purified using a Ni^2+^-NTA purification kit according to the product instructions. The BCA Protein Assay Kit to check the concentration of protein (Beyotime P0012).

One unit of MoOxdC activity is defined as the amount of enzyme required to convert 1 μmol of substrate to product in 1 min [13,71]. For the ability of MoOxdC to degrade oxalic acid, refer to the method of Svedruzˇic´ et al. and made some changes [44]. The activity of oxalate decarboxylase was determined by using oxalic acid (Macklin reagent, CAS: 144-62-7) as substrate. The enzyme reaction system is as follows: add 0.2 mL 8 mm oxalic acid solution, 1.2 mL 50 mmol/L pH 4.0 citrate buffer solution in the tube and put it at 37°C preheat 2 min, respectively. Then add 0.1 mL different concentrations of MoOxdC protein (0.15, 0.3, 0.45, 0.6 mg/mL) solution. Heat protein solutions with concentrations 0.1 and 0.6 mg/mL at 100°C for 10 minutes to inactive them, and use the inactivated enzyme solution as a control. The reaction solution is placed at 37°C for the reaction. The reaction solution was terminated by adding an equal volume of 0.2 mol/L potassium dihydrogen phosphate after 30 min. The remaining oxalic acid in the reaction solution was detected by spectrophotometry (describe in the next paragraph) to calculate the activity of oxalate decarboxylase, and the formic acid content produced in the reaction solution was determined by HPLC, indicating that oxalate decarboxylase specifically degrades oxalic acid into formic acid. Regarding the experiment on the effect of metal ions on MoOxdC enzyme activity, different concentrations of Mn^2+^ (MnCl_2_, 0.05, 0.1, 0.2, 0.3, 0.4 mM) and Fe^2+^ (FeSO_4_, 0.05, 0.1, 0.2, 0.3, 0.4 mM) were added in the enzyme reaction system (contains 0.1 mL 0.6 mg/mL enzyme solution) to verify the effect of metal ions on MoOxdC enzyme activity.

The residual amount of oxalic acid in the reaction solution was determined by the method of Wang et al. [72]. Next, 90 μL of oxalic acid at different concentrations and add to the premix solution (100 μL 0.5 mg/mL FeCl_3_ solution, 1 mL 0.2 mol/L KCl buffer solution pH = 2, and 60 μL 0.5 % sulfosalicylic acid) and color development at 28 °C for 30 min, and the absorbance value was measured at 510 nm. The samples were colored and measured using the aforementioned method, and the oxalic acid content in the samples was determined according to the standard curve. Oxalic acid solution (2 mg/mL) was successively diluted to final concentrations of 0.055, 0.111, 0.222, and 0.444 mg/mL. Formic acid content was measured using an Agilent 1260 Infinity II LC System equipped with a VWD detector. The check wavelength of the detector was 210 nm. The moving phase was 10% methanol (Sigma-Aldrich 34,860-4 L-R, suitable for HPLC, water containing 0.1% phosphoric acid). The loading quantity of sample was 100 μL. The concentration of formic acid was calculated by converting the peak area of formic acid into a standard curve.

### Construction of knockout mutants, complementary strain and subcellular localization assay

The knockout vector pCX62, used to delete the *MoOxdC* gene in *M. oryzae* was constructed using a split-marker approach [73]. The 0.9 kb upstream (A) and 0.7 kb downstream (B) regions were amplified using primers MoOxdC-AF/MoOxdC-AR and MoOxdC-BF/MoOxdC-BR, respectively. The resulting PCR products were cloned into the Kpn I-EcoR I and BamH I- Sac I sites of vector pCX62 respectively using ClonExpress II One step Clong Kit (Vazume, C112), and following the manufactures’ guidelines. Then two vectors A-pCX62 and pCX62 were obtained. The AH (A fragment+HYG) fragment was amplified with primers MoOxdC-AF and MoOxdC-UAR, and the BH (HYG+B fragment) was amplified with primers MoOxdC-UAR and MoOxdC-BR (Fig. S2a). Then the two PCR products were recovered by the SanPrep Column PCR Product Purification Kit (Sangon Biotech, B518141). Using the PEG-mediated protoplast transformation method, targeted deletion using a homologous recombination approach was used []. Both the AH and BH fragment were transferred into *M. oryzae* protoplasts and grown in TB3 medium containing 300 mm hygromycin resistance. After 5 to 7 days of culture at 28 °C, transformants were selected and verified. The transformants were verified using ORF (MoOxdC-OF, MoOxdC-OR), UAH (MoOxdC-UAF, MoOxdC-UAR), and another gene (MGG_15997, 15997-OF, 15997-OR). This method allows for rapid assembly of deletion and efficient targeted integration of these constructs via protoplast transformation [74]. Moreover, the frequence of correctly targeted deletion constructs in fungal systems is potentially increased because only transformants in which the two overlapping marker fragments have successfully recombined will grow in selective medium [74]. The same method to knockout *Moalo1*. The 0.98 kb upstream and 1.1 kb downstream regions were amplified, respectively. The transformants were verified by ORF, UAH and HYG.

Subcellular localization was first predicted using ProtComp 9.0, an online website (http://www.softberry.com/berry.phtml?topic=protcompan&group=programs&subgroup=proloc). The complementary vector pKD7-Red was used for the complementary strain gain. The cDNA fragment of MoOxdC was located between the Xba I-Sal I sites of the vector pKD7-Red, and a MoOxdC-pKD7-Red complementary vector was constructed. The MoOxdC-pKD7-Red complementary vector was then transferred into the Δ*Mooxdc* mutant to obtain the complementary strain Δ*Mooxdc/oxdc*. Confocal fluorescence microscopy (Olympus Fluoview FV3000, Olympus) was used to observe the fluorescence of mycelium and conidia of Δ*Mooxdc/oxdc*, while the mycelium and conidia were stained with mitochondrial MitoTracker Green dye (Beyotime, C1048). If RFP and MitoTracker Green were co-located, it indicated that MoOxdC was located in the mitochondria.

### Oxalic acid degradation experiment

Oxalic acid solution (suction filter sterilization) at a concentration of 0.09 g/L was added to PDA medium at pH 7 to the oxalic acid final concentrations of 0, 2, 4, and 6 mM, 0.05 % bromophenol blue was added to PDA medium as an indicator. Then strains were inoculated into the medium and cultured at 28 °C. After culturing for 7 days, the colony diameter and inhibition rate were measured. This experiment was based on the method of Zeng et al. [15]. Similarly, oxalic acid (0.09 g/L) was added to the liquid PD medium to obtain oxalic acid final concentrations of 0, 2, 4, and 6 mM. and The strains were incubated at 150 rpm, 28 °C for 7 days, and the content of oxalic acid in the culture medium was determined. Oxalic acid content was determined following the method described by Wang et al. [75], as described above. For the feedback inhibition response, formic acid was added to the reaction system at a final concentration of 1 mm. The other steps were consistent.

### Analysis of MoOxdC on the growth, conidiation, conidial germination and appressorium formation

The mutants and wild-type cake with a diameter of 0.5 cm were inoculated on CM and PDA medium, respectively. The colony diameter was measured after culturing for 7 days. The diameter of each plate was measured 3 times at different locations centered on the strain cake, with three replicates per group, and the average value was calculated. When the strains were cultured on RBM after 10 days, a sterile cotton swab was used to hang off the surface mycelium, and a piece of the culture medium (0.5 × 4 cm) was cut with a blade and placed on a glass slide. It was then placed in a moisturizing box and incubated at 28 °C. The prepared sample was used to observe the production of conidiophores under a Nikon Eclipse 80i microscope at 24, 48 and 72 h. The conidiophores were stained with lactophenol cotton blue to count the number of conidiophores. The mycelium will be stained blue, while the conidiophores and conidia will not be stained, so the conidiophores and mycelium can be distinguished and counted. The number of conidiophores in a field of vision under a 20-fold mirror was counted. Three pieces were cut for each strain, and each piece was counted three times at different vision, and the average value was calculated[]. The Conidia were collected with 2 mL sterile water when the plates were cultured under light and 28 °C for 3 days after hanging off the mycelium. The number of conidia was counted by blood cell counting plate. Conidia were diluted with 0.25 % gelatin to 5 × 10^4^ conidia/mL. The 20 μL conidial suspension was added dropwise to a hydrophobic cover slip. Each group had three replicates, and each replicate was counted for three times. Then the hydrophobic slide was placed in a wet box and cultured at 28 °C. Then the conidial germination and appressorium formation were observed under the microscope after culture 4, 8, 12 and 24 h. The conidial germination rate was (the number of germination per 100 conidial/100)×100%. The appressorium formation rate was (the number of appressorium formation 100 conidial/100)×100%.

### Pathogenicity assays

The rice used for the pathogenicity assay was “*Oryza sativa* cv. Lijiangxintuanheigu.” A 1 mL conidia solution prepared according to the method in section 4.6 was inoculated on the fourth leaf stage rice leaves. For point inoculation, one point was 20 μL, and three dots were placed on each rice leaf. The inoculated plants were placed in the dark in a dew chamber (humidity 50%) for 24 h at 28 °C and then transferred to a growth chamber (humidity 50%) with a photoperiod of 16 h for 5 days post-inoculation (dpi). The lesion area was counted using ImageJ software. The conidia solution with and without 1 mm formic acid was injected into the leaf sheaths of 3-weeks-old rice plants and placed in the dark in a dew chamber (humidity 50%) at 28 °C. Leaf sheath infection was observed under a microscope at 12, 24, and 48 h. The infection rate was measured using the different types of infections. Type I is the formation of an appressorium, Type II is the first rice cell infection, and Type III is the infection of second rice cells or more cells. These experiments were performed with three independent replicates, and the representative results from one of these experiments are presented.

### H_2_O_2_ stress assay, fatty acid utilization assay, acetyl CoA content and ATP content determination

When the mutant and wild-type strains were grown on CM medium for 7 days, they were inoculated on CM medium containing different concentrations of H_2_O_2_ (2.5, 5 and 7.5 mM). The diameter of the colonies was measured after seven days of growth. The H_2_O_2_ content in the mycelia was determined using a Hydrogen Peroxide (H_2_O_2_) Content Assay Kit (Sangon Biotech, D799774–0100).

For the fatty acid utilization assay, the wild type and mutant were inoculated on MM media containing 1 % (w/v) glucose, 2% (v/v) Ole oil, 1 mm sodium laurate or 1 mm sodium acetate as the sole carbon source. Ole oil was dissolved in 0.03 % Tween 80 (v/v), and 0.03 % Tween 80 (v/v) was added to MM as a control of Ole oil [42]. ATP production was measured using an ATP Bioluminescence Assay Kit (Beyotime; S0026). Acetyl CoA content was measured using an Acetyl Co-A Content Assay Kit (Solarbio, BC0980).

### Quantitative real-time PCR

For gene expression analysis, total RNA was isolated from mycelia using RNAiso Plus (TaKaRa, 9109). First-strand cDNA was synthesized by reverse transcription-polymerase chain reaction (RT-PCR) using the GenStar Reverse Transcription Kit (Genestar, A214–10). The reverse-transcribed cDNA was used for quantitative real-time PCR using an Applied Biosystems 7500 Real-time PCR system and SYBR® Premix EX Taq^TM^ ІІ (TaKaRa, RR420). The relative mRNA levels were calculated using the 2^−ΔΔCt^ method. The primers used are listed in Supplementary Table 2.

### Statistical analysis

All experiments were performed at least three times. The significance of the data was assessed using an independent-samples t-test and one-way analysis of variance. The threshold for statistical significance was *p* < 0.05.

## Supplementary Material

Supplementary Figure 6.tif

Supplementary Figure 1.tif

Supplementary Table 1.docx

Supplementary Figure 5.jpg

Supplementary Table 2.docx

Supplementary Figure 4.jpg

Supplementary Figure 2.tif

Supplementary Figure 3.tif

## Data Availability

All data generated or analyzed during this study are deposited in Figshare, DOI: https://doi.org/10.6084/m9.figshare.26531719.
