# Supplementary figures and images for: Decarboxylase mediated oxalic acid metabolism is important to antioxidation and detoxification rather than pathogenicity in Magnaporthe oryzae
Source: Virulence. 2025 Jan 15;16(1):2444690. doi: 10.1080/21505594.2024.2444690 (PMC11776485; doi:10.1080/21505594.2024.2444690)

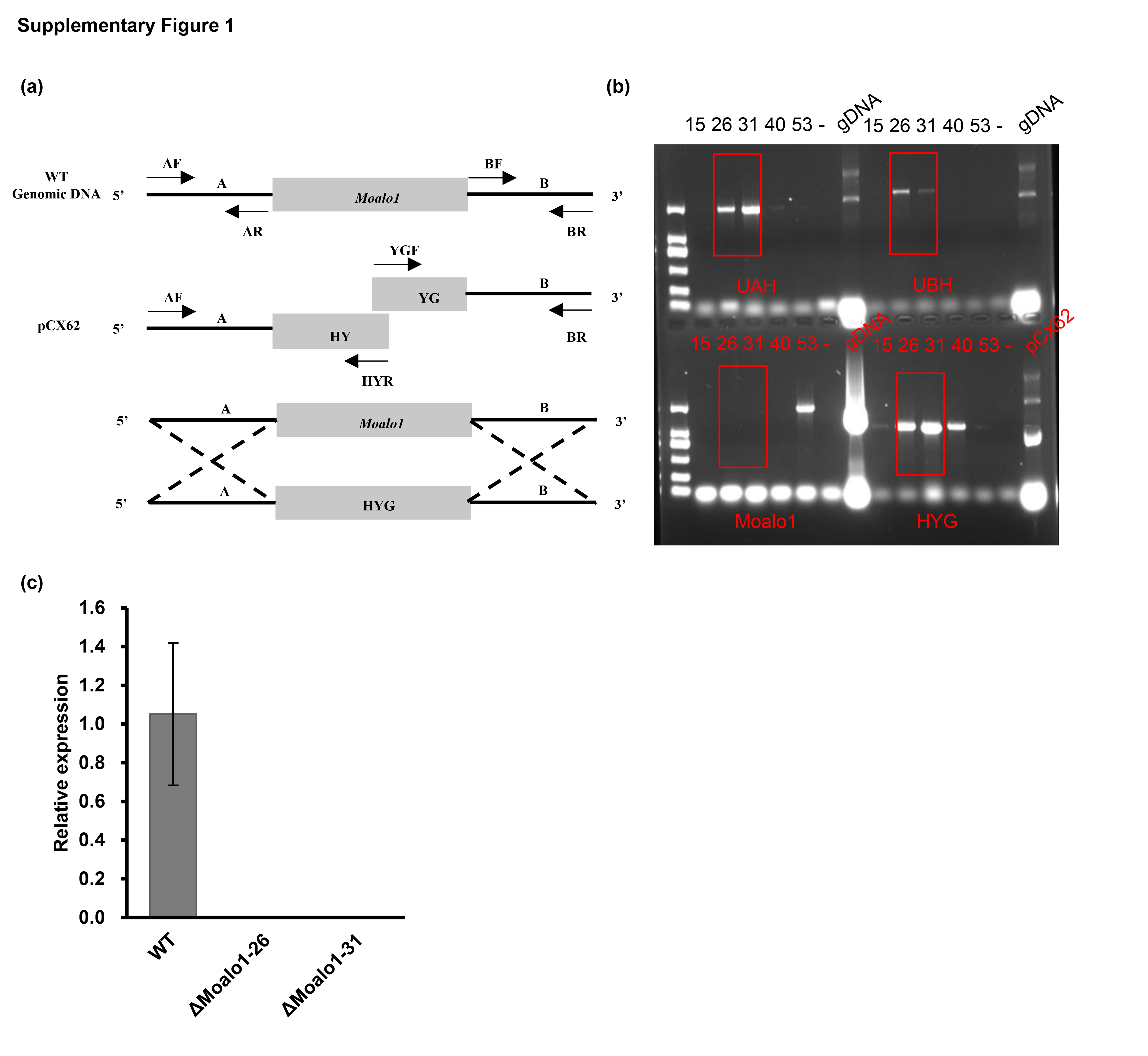

Supplement: Supplementary Figure 1.tif [file KVIR_A_2444690_SM3300.tif]

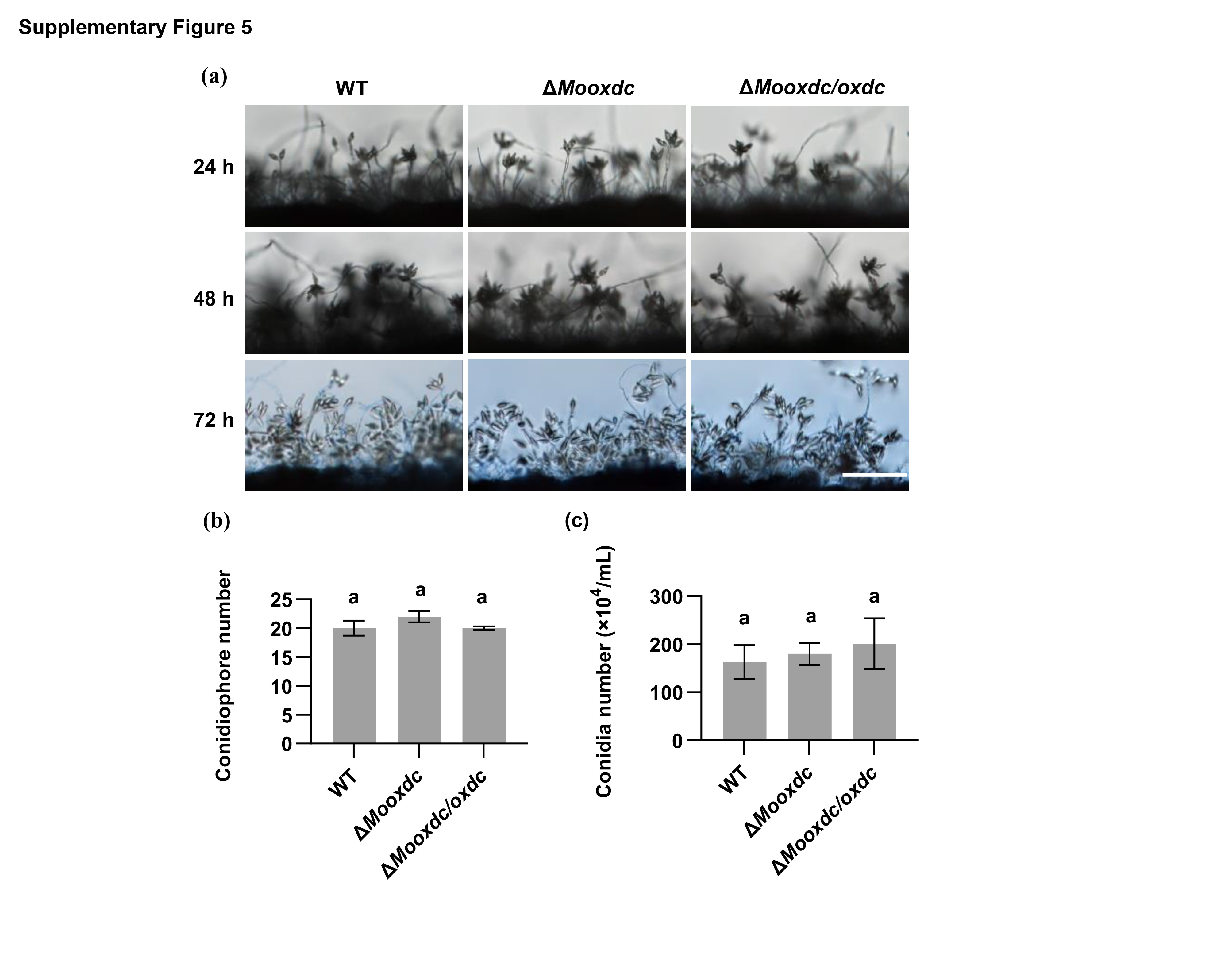

Supplement: Supplementary Figure 5.jpg [file KVIR_A_2444690_SM3298.jpg]

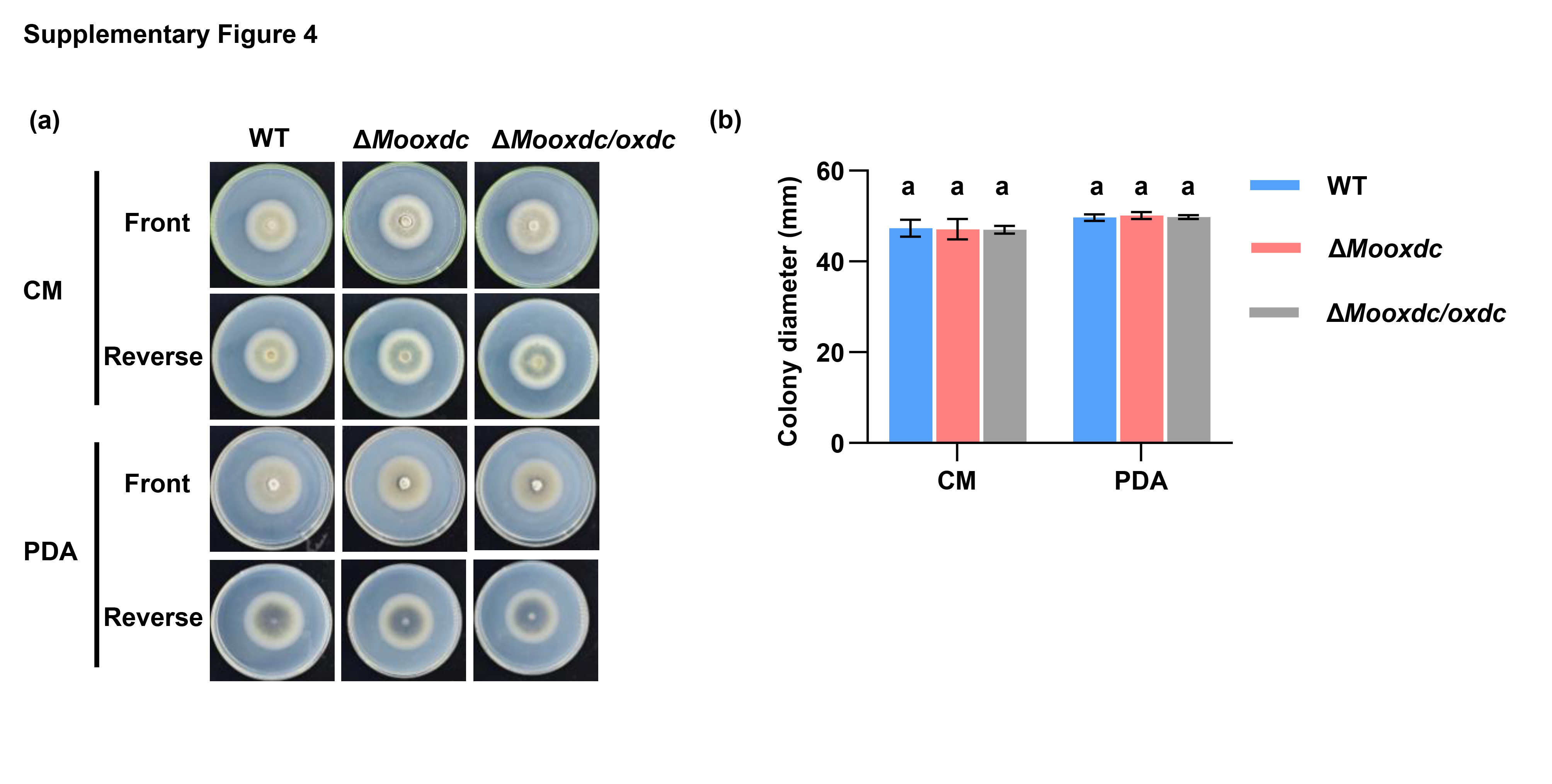

Supplement: Supplementary Figure 4.jpg [file KVIR_A_2444690_SM3296.jpg]

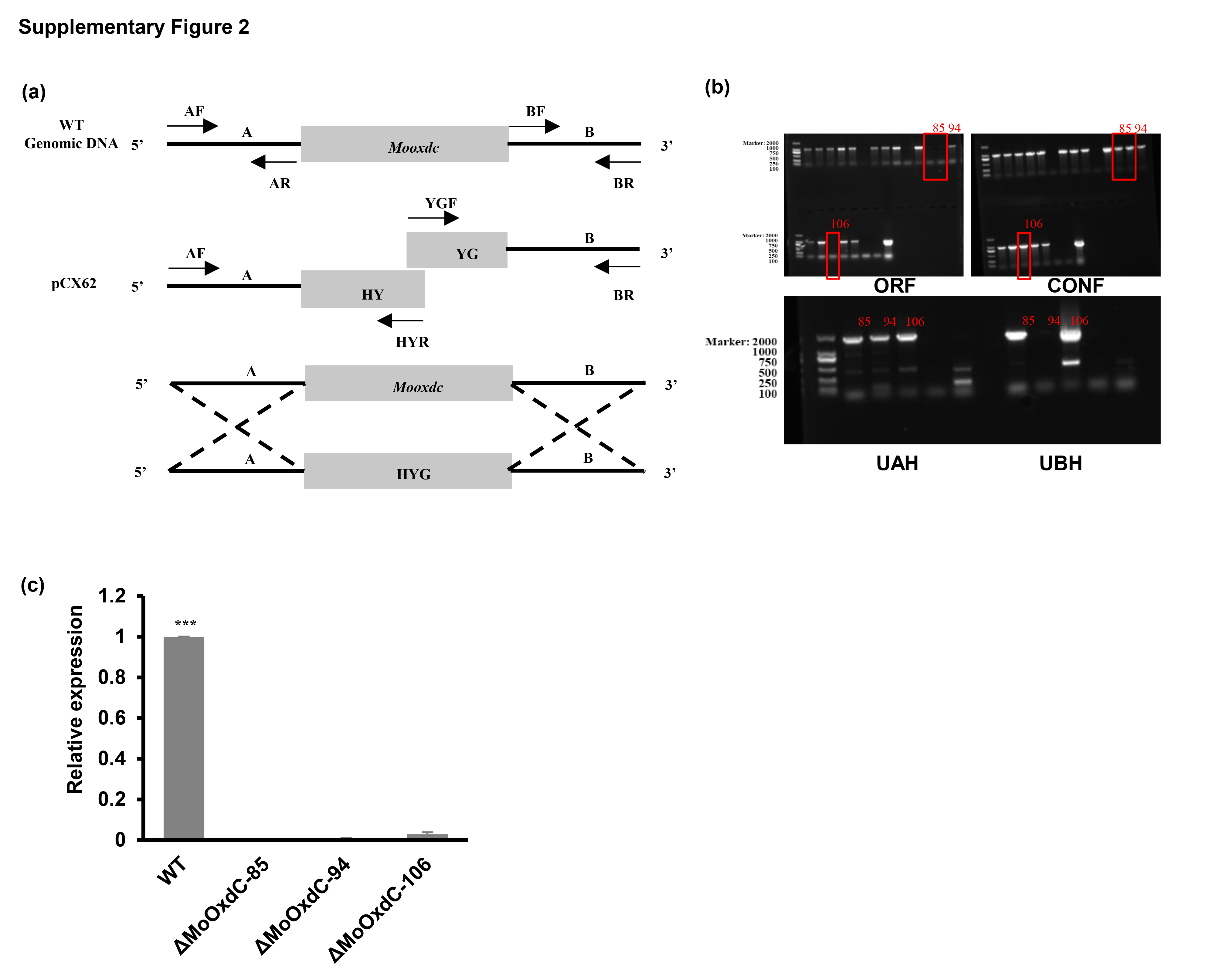

Supplement: Supplementary Figure 2.tif [file KVIR_A_2444690_SM3295.tif]

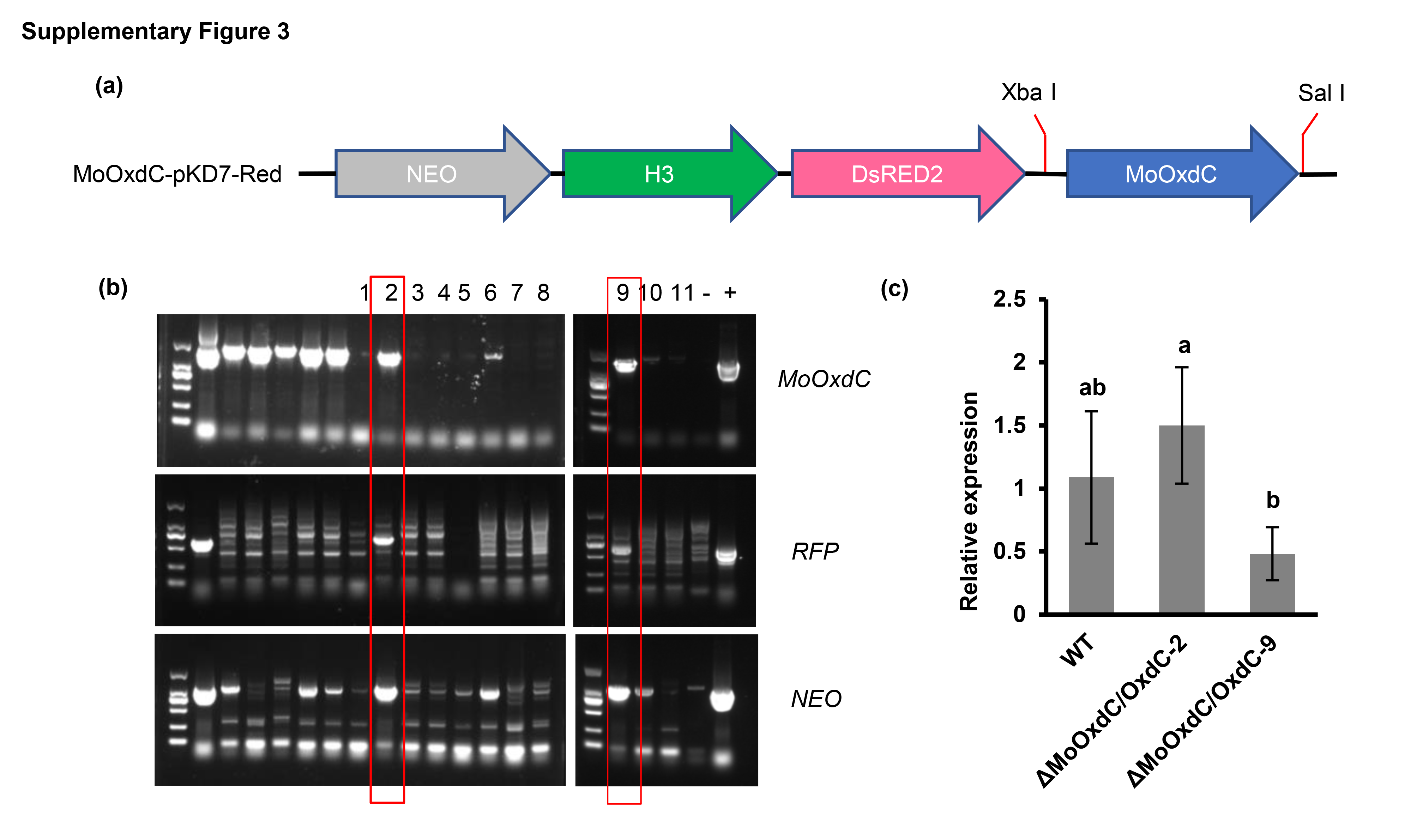

Supplement: Supplementary Figure 3.tif [file KVIR_A_2444690_SM3294.tif]
