# Supplementary material for: Decarboxylase mediated oxalic acid metabolism is important to antioxidation and detoxification rather than pathogenicity in Magnaporthe oryzae
Source: Virulence. 2025 Jan 15;16(1):2444690. doi: 10.1080/21505594.2024.2444690 (PMC11776485; doi:10.1080/21505594.2024.2444690)
Supplement: Supplementary Table 1.docx [file KVIR_A_2444690_SM3299.docx]

**Table S1. Identifying subcellular location of genes in oxalic acid synthesis, metabolism and transport pathway.**

| **Prediction of subcellular localization** | | |
| --- | --- | --- |
| **Gene ID** | **Subcellular localization** | **Score** |
| MGG_14061 (MoOxdC) | Mitochondrial | 4.9 |
| MGG_10252 (MoOxO) | Mitochondrial | 4.1 |
| MGG_09838 (MoOxT1) | Endoplasmic reticulum/ Plasma membrane | 9.9/2.5 |
| MGG_15133（MoOxT2） | Plasma membrane | 10 |
| MGG_02689 (MoALO1) | Cytoplasmic/ Mitochondrial | 3.1/2.4 |
| MGG_06981 (MoCRAT2) | Mitochondrial/ Peroxisomal | 9.7/2.7 |
| MGG_01721 (MoPTH2) | Mitochondrial | 9.9 |

Note: Score is the Integral Prediction of protein location.
