# Supplementary material for: Decarboxylase mediated oxalic acid metabolism is important to antioxidation and detoxification rather than pathogenicity in Magnaporthe oryzae
Source: Virulence. 2025 Jan 15;16(1):2444690. doi: 10.1080/21505594.2024.2444690 (PMC11776485; doi:10.1080/21505594.2024.2444690)
Supplement: Supplementary Table 2.docx [file KVIR_A_2444690_SM3297.docx]

| **Primer name** | **Sequence (5’-3’)** | **Remark** |
| --- | --- | --- |
| MoOxdC-AF | TCGCTATGATGGCGTTGAG | Amplify *MoOxdC* up flank sequence |
| MoOxdC-AR | GGATGGGATTCGGGATTTA |  |
| MoOxdC-BF | TTTTACTCGGGTTGGTGG | Amplify *MoOxdC* down flank sequence |
| MoOxdC-BR | CCTATCCTGGCGTTACATTTT |  |
| MoOxdC-OF | CCTCTTTGCTCCTCCCACC | Verify *MoOxdC* ORF fragment |
| MoOxdC-OR | ACACGCTAATGTCGCTGAACT |  |
| MoOxdC-QF | CCCTGAGCCACAACAAGC | qRT-PCR primer of *MoOxdC* |
| MoOxdC-QR | TGTGGTTCTTCCCGCCC |  |
| MoOxdC-UAF | GTCTGTGCGTGCCTAAAA | Verify *MoOxdC* knockout mutant sequence UAH |
| MoOxdC-UAR | GCTCCATACAAGCCAACC |  |
| MoOxdC-UBF | AACTCACCGCGACGTCTGTC | Verify *MoOxdC* knockout mutant sequence UBH |
| MoOxdC-UBR | GCTCCATACAAGCCAACC |  |
| Moalo1-AF | CGCGAGATACTAAAAAGAATCT | Amplify *Moalo1* up flank sequence |
| Moalo1--AR | ACTGGGTAAGATCGGACGT |  |
| Moalo1--BF | ACGGAACTTTGCAGAGAAT | Amplify *Moalo1* down flank sequence |
| Moalo1--BR | GCATCAGACCTCGCATGCT |  |
| Moalo1-OF | ATGCGACGTAACAAATCAAG | Verify  *Moalo1* ORF fragment |
| Moalo1-OR | TTTTCTGCCTCCGCAGTCC |  |
| Moalo1-UAF | CGCCGAGTATGACGGCAT | Verify  *Moalo1* knockout mutant sequence UAH |
| Moalo1-UAR | GCTCCATACAAGCCAACC |  |
| Moalo1-UBF | AACTCACCGCGACGTCTGTC | Verify  *Moalo1* knockout mutant sequence UBH |
| Moalo1UBR | GAAAGCAGTTGGTCTCAAGT |  |
| MoOxdC-PE-F | CGGAATTCATGAGACAATTCATTGCAGG | Protein expression primer of *MoOxdC* |
| MoOxdC-PE-R | CCGCTCGAG TCGGCGCTTGGTGGCGGTCG |  |
| MoOxdC-Com-F | GCTCTAGAATGAGACAATTCATTGCAGG | Amplify *MoOxdC* complementation sequence |
| MoOxdC-Com-R | GCGTCGACTCGGCGCTTGGTGGCGGTCG |  |
| 02689-QF | ATGCGACGTAACAAATCAAGCGA | qRT-PCR primer of *Moalo1* |
| 02689-QR | TCTTCTCGATTTCGGCCTCGGA |  |
| 01721-QF | CTCGCTGCCAGCAGGCTAT | qRT-PCR primer of *Mopth2* |
| 01721-QR | TGGTGCTGTTTGCCAGCTC |  |
| 06981-QF | TCGACGACGAACAACGCC | qRT-PCR primer of *MoCrat2* |
| 06981-QR | CGTAGCCGCCAAGCTCGT |  |
| 09838-QF | CGACAGTGTGCTTTCGCG | qRT-PCR primer of *MoOxT1* |
| 09838-QR | TCTCTCGCAACCATAATGACAGT |  |
| 15133-QF | CGCCATTTTGATCTTGCACGT | qRT-PCR primer of *MoOxT2* |
| 15133-QR | AGCCTGTTGGCACAGGG |  |
| 10252-QF | CTCGGTCAACATGCGCCTC | qRT-PCR primer of *MoOxO* |
| 10252-QR | GCTGGGGAAGTACCAGAGGTC |  |
| MoActin-S | CGTTGTTCCTATTTACGAGGG | qRT-PCR primer of *MoActin* |
| MoActin-A | TTGATGTCACGGACGATTTC |  |
| 15997-OF | CAGAATCCCACCACAGAAA | Verify MGG_15997 ORF fragment |
| 15997-OR | GCCATATTAGTCGCCTTGA |  |

**Table S2. Primers used in this study**
